# Perceptions, Attitudes, and Barriers to Obesity Management in Spain: Results from the Spanish Cohort of the International ACTION-IO Observation Study

**DOI:** 10.3390/jcm9092834

**Published:** 2020-09-02

**Authors:** Javier Salvador, Nuria Vilarrasa, Francisco Poyato, Miguel Ángel Rubio

**Affiliations:** 1 Department of Endocrinology and Nutrition, University Clinic of Navarra, 31008 Pamplona, Spain; 2 CIBER Physiopathology of Obesity and Nutrition (CIBERobn), 28029 Madrid, Spain; 3 Department of Endocrinology, Diabetes and Nutrition, Bellvitge University Hospital-IDIBELL, L’Hospitalet de Llobregat, 08907 Barcelona, Spain; nuriavilarrasa@yahoo.es; 4 CIBER Diabetes and Associated Metabolic Diseases (CIBERDEM), 28029 Madrid, Spain; 5 Obesity Medical Department, Novo Nordisk Spain, 28033 Madrid, Spain; fpca@novonordisk.com; 6 Department of Endocrinology and Nutrition, San Carlos Clinical Hospital and Health Research Institute of the San Carlos Clinical Hospital (IdISSC), 28040 Madrid, Spain; marubioh@gmail.com; 7 Faculty of Medicine, Complutense University of Madrid, 28040 Madrid, Spain

**Keywords:** obesity, weight loss, ACTION-IO, perceptions, barriers, obesity management, Spain

## Abstract

The prevalence of obesity is rapidly rising in Spain. The Awareness, Care and Treatment in Obesity Management—An International Observation (ACTION-IO) study (NCT03584191) was an international cross-sectional survey conducted to identify the perceptions, attitudes, behaviors, and barriers to obesity management for people with obesity (PwO) and healthcare professionals (HCPs); results from Spain are presented. In Spain, 1500 PwO (body mass index ≥30 kg/m^2^ based on self-reported height and weight) and 306 HCPs (in direct patient care for ≥2 years) completed the survey. Fewer PwO (59%) than HCPs (93%) agreed that obesity is a chronic disease. Most PwO (80%) assumed complete responsibility for their own weight loss, whilst 19% of HCPs placed the responsibility on PwO. One-fifth of PwO stated they began struggling with weight before age 15. The mean delay in discussing weight for the first time with an HCP was 6 years. Only 24% of HCPs thought their patients were motivated to lose weight, whilst 45% of PwO reported being motivated. Of the 67% of PwO who had discussed their weight with an HCP in the last 5 years, 66% had been formally diagnosed with obesity. Our Spanish dataset reveals discrepancies in the perceptions and attitudes between PwO and HCPs, thus highlighting the need to improve education about obesity and its clinical management.

## 1. Introduction

The current obesity pandemic is among the main challenges for public health due to its impact on early morbidity and mortality and linked associated comorbidities [1,2]. Excessive adipose tissue, particularly visceral and ectopic fat, represent independent predictive factors of major acute cardiovascular events and mortality [3]. In addition, obesity reduces the patient quality of life and is a major burden on healthcare and healthcare system expenses [4]. Most recently, obesity has also been identified as a potential key risk factor for susceptibility to COVID-19 [5,6].

The prevalence of obesity in Spain is 22.9% and 39.4% of individuals are considered overweight [7], with an upward trend predicted in the coming years, as seen in other Western countries [8]. In fact, if the current trend continues in Spain, it has been estimated that the number of patients with excess weight will increase by 16% by 2030, leading to 3.1 billion EUR per year of extra direct medical costs [9].

Obesity is a complex, chronic disease with a multifactorial etiology; genetic, epigenetic, physiological, behavioral, socio-cultural, and environmental factors all contribute to the imbalance in energy homeostasis that leads to obesity [10,11,12]. Guidelines defined by different scientific societies are currently available to help effectively address the diagnosis and treatment of obesity [13,14,15]. However, healthcare professional (HCP) familiarity with these guidelines and knowledge of evidence-based clinical management of obesity is reportedly low [16,17]. Furthermore, people with obesity (PwO) are often subject to stigma and discrimination in both social and healthcare settings [18,19]. Discrimination and stigma (including self-directed stigma whereby PwO blame themselves) is associated with poor mental health, poor quality of life, unhealthy eating, and avoidance of exercise among others [19,20,21]. Obesity stigma can exist among HCPs, which poses barriers to the diagnostic and therapeutic approach to this condition [18], and is associated with poor treatment outcomes for PwO [19]. Evidence suggests that a lack of education regarding the complex etiology of obesity contributes to obesity stigma [19]. Due to the public health threat that obesity stigma poses, guidelines and an international consensus of experts have called for ending obesity stigma via increased education and better communication between HCPs and PwO in order to address the barriers to diagnosis and treatment [15,18,19].

The Awareness, Care and Treatment In Obesity maNagement (ACTION) studies were designed to identify the perceptions, attitudes, behaviors, and potential barriers to obesity management for PwO and HCPs. The results may provide an awareness of the real problem and the difficulties encountered by both groups in the approach and control of obesity. Results are currently available from the ACTION US [22], ACTION Canada [23], and ACTION—An International Observation (ACTION-IO) [24] studies, with Spain participating in ACTION-IO. The main objective of this paper is to present the results obtained from the Spanish population, highlighting the similarities and differences with respect to the global results from all participating countries, in order to establish specific strategies to optimize the treatment of obesity in Spain.

## 2. Methods

### 2.1. Study Design and Participants

The methodology for the ACTION-IO study has been reported previously [24]. In summary, it was a cross-sectional, non-interventional, descriptive study that collected data via an online survey of PwO and HCPs conducted in 11 countries: Australia, Chile, Israel, Italy, Japan, Mexico, Saudi Arabia, South Korea, Spain, the United Arab Emirates (UAE), and the United Kingdom (UK). Spanish participants completed the survey between July 27, 2018 and September 6, 2018.

The study was designed by an international steering committee of obesity experts, representing primary care, medical specialties, and psychology, and three medical doctors employed by Novo Nordisk. The survey was based on those used in the ACTION US [22] and ACTION Canada [23] studies. To avoid response bias, questionnaire items were carefully phrased and presented in the same order and the listed items were shown in alphabetical, categorical, chronological or random order, as relevant for each response set, the full text of the questionnaires has been previously published [24]. A third-party vendor (KJT Group (Honeoye Falls, NY, USA)) conducted the online survey and managed the data collection and analysis. In Spain, the study questionnaires were approved by the Research Ethical Committee of the University of Navarra (Pamplona, Spain). All procedures in this study complied with the laws and regulations regarding the management of personal information as required by Spain and the European General Data Protection Regulation. The study was sponsored by Novo Nordisk, conducted in accordance with the Guidelines for Good Pharmacoepidemiology Practices [25] and the Declaration of Helsinki [26], and is registered with ClinicalTrials.gov (NCT03584191).

Eligible PwO were aged 18 years or older, with a current body mass index (BMI), based on self-reported height and weight, of at least 30 kg/m^2^. Exclusion criteria included pregnancy, participation in intense fitness or body building programs, or significant, unintentional weight loss during the past 6 months. Eligible HCPs were medical practitioners who had been in practice for 2 years or more, with at least 50% of their time involved in direct patient care and who had seen 100 or more patients during the past month, at least ten of whom had a BMI of at least 30 kg/m^2^. HCPs specializing in general, plastic, or bariatric surgery were excluded. All respondents provided electronic informed consent prior to initiation of the screening questions and survey.

Respondents were mostly recruited via online panel companies to whom they had given permission to be contacted for research purposes. Spanish respondents were recruited via email and were offered the survey in Spanish or English. To minimize the sampling bias, PwO data were stratified, whereby the outbound sample was sent according to pre-determined demographic targets based on age, gender, household income, education, and region. Targets were established based on data from the Organization for Economic Co-operation and Development (Labour Force Survey, 2018) and the US Census Bureau, International Data Base, and were monitored throughout the data collection to ensure population representativeness. Prior to participation, PwO were blinded to the specific study goals, being informed that the purpose was “to determine the treatment experiences of patients with a specific condition”. Screening questions were used to determine eligibility based on these demographic targets. Respondents who passed the screening process, had a BMI of at least 30 kg/m^2^, and who met the other study eligibility criteria, were permitted to complete the full survey.

Sample sizes were calculated based on the usual acceptance of a smallest sub-sample (*n* ~ 50), previous experience with the ACTION US and ACTION Canada studies, and the intention to conduct country-specific sub-analyses. Response rates for the international ACTION-IO study were expected to be ~9.7% for PwO and ~20.4% for HCPs, based on what was seen in ACTION US. A statistical analysis was not conducted to determine the sample size, but rather market knowledge, previous market research experience, published data on the prevalence of obesity in Spain, and total population estimates were utilized to determine the sample feasibility and requirement. Given these inputs, a sample of size 1500 for PwO and 300 for HCPs was determined optimal. Additional PwO sample size considerations for the Spanish cohort of the ACTION-IO study are presented in Table 1.

### 2.2. Statistical Analysis

The analysis of de-identified data was performed by the KJT Group using SPSS (IBM, version 23.0), Stata (StataCorp LLC, version IC 14.2), and Excel (Microsoft, version 2016). Results were presented using descriptive statistics (means, medians, frequencies). The final PwO sample, which satisfied the representative demographic targets (age, gender, household income, education, and region), was weighted. The HCP data were monitored for specialist types but were not weighted.

## 3. Results

### 3.1. Participants

The ACTION-IO survey was completed by 1500 PwO and 306 HCPs in Spain (Table 2). The response rate was 24% for PwO and 6% for HCPs, with final eligibility rates of 11% and 55% for each group, respectively. Approximately half (52%) of the PwO were female and the majority had a BMI classification of Class I obesity (BMI 30.0–34.9 kg/m^2^); the mean age was 45 years. Of the participating Spanish HCPs, almost half were specialists, with 24% of HCPs specializing in endocrinology.

### 3.2. Perception of Obesity as a Chronic Disease

A total of 93% of HCPs considered obesity to be a chronic disease. Despite 83% of PwO reporting one or more comorbidities, only 59% considered obesity to be a chronic disease. Most PwO (80%) and HCPs (86%) considered obesity to have a large impact on overall health, like other chronic diseases, such as stroke, diabetes, cancer, or chronic obstructive pulmonary disease (Supplementary Figure S1). However, PwO tended to underestimate their own weight status: 73% considered themselves as having either normal weight or being overweight, while the remaining PwO perceived themselves as having obesity (24%) or extreme obesity (2%).

### 3.3. Responsibility and Motivation for Treating Obesity

**Figure 1:**
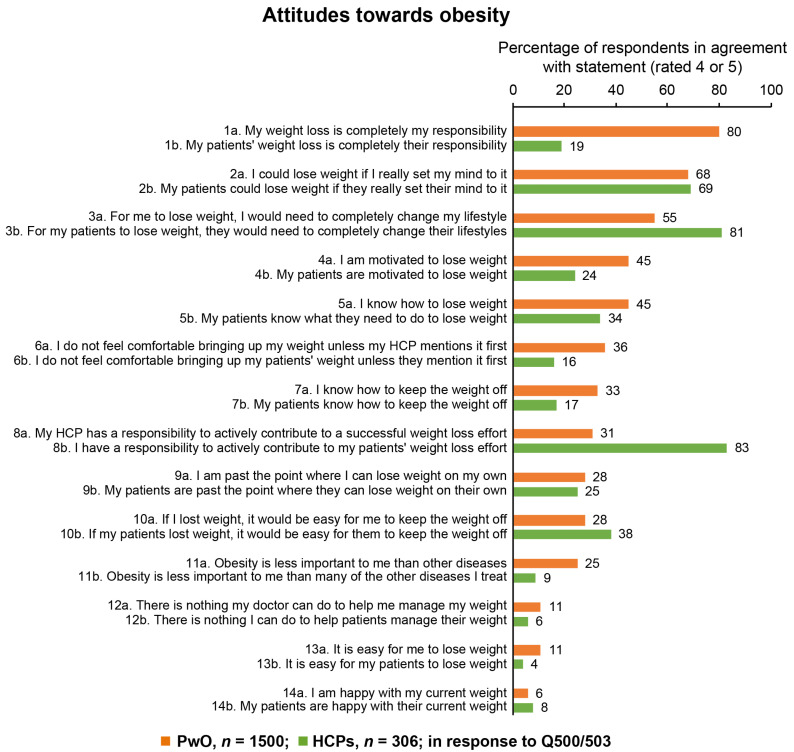
PwO and HCP agreement with statements regarding attitudes towards obesity. PwO = orange; HCPs = green. HCP, healthcare professional; PwO, people with obesity. Rated on a scale of 1–5.

The majority of PwO (80%) assumed complete responsibility for their own weight loss and 68% believed they could lose weight if they tried (Figure 1). Conversely, only 19% of HCPs considered weight loss to be the sole responsibility of their patients with obesity; most HCPs (83%) also considered their patient’s weight loss to be a responsibility of their professional practice. Only 24% of HCPs believed that their patients were motivated to lose weight, in contrast with the 45% of PwO who stated that they were, in fact, motivated to lose weight. Both PwO and HCPs perceived unhealthy eating habits (56% of PwO; 90% of HCPs) and lack of physical activity (70% of PwO; 90% of HCPs) as barriers for weight loss, whereas fewer than half (39% of PwO; 42% of HCPs) considered genetic factors to be an obstacle.

### 3.4. Previous Weight Loss Attempts and Outcomes

**Figure 2:**
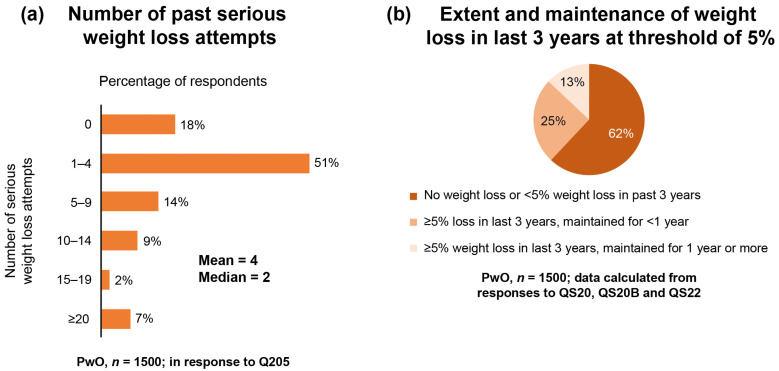
Weight loss efforts and maintenance. (**a**) Number of serious past weight loss attempts by PwO; (**b**) PwO extent and maintenance of weight loss in the last 3 years at a threshold of 5% of total body weight. PwO, people with obesity.

Most PwO reported having made at least one serious attempt at weight loss (mean: four attempts) (Figure 2a); however, only 13% of PwO were able to maintain a weight loss of ≥5% for at least one year (Figure 2b).

### 3.5. Conversations Between PwO and HCPs

**Figure 3:**
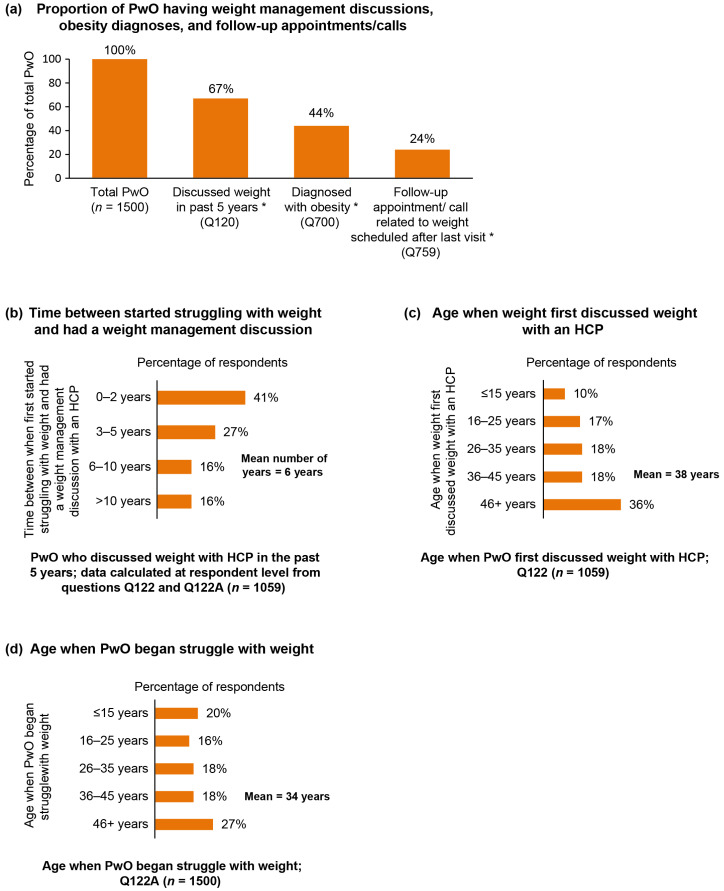
Weight management conversations and outcomes. (**a**) The proportion of PwO who have had weight management discussions with an HCP, obesity diagnoses, and follow-up appointments/calls; (**b**) time gap between first struggling with weight and having a weight management discussion with an HCP; (**c**) the age when PwO first discussed their weight with an HCP; (**d**) the age when PwO began their struggle with weight. * Percentage results are based on the total number of Spanish PwO (*n* = 1500). HCP, healthcare professional; PwO, people with obesity.

Only 67% of all PwO reported having had a conversation with their HCP about their weight problem in the past 5 years (Figure 3a). Surprisingly, the mean time interval between the initial concern regarding weight and the first conversation was 6 years (Figure 3b) and the mean age at the time of the initial consultation was 38 years (Figure 3c). Although the mean age at which PwO began struggling with their weight was 34 years, there was a high percentage of PwO whose concerns about excess weight began at under 16 years of age (20%; Figure 3d).

**Figure 4:**
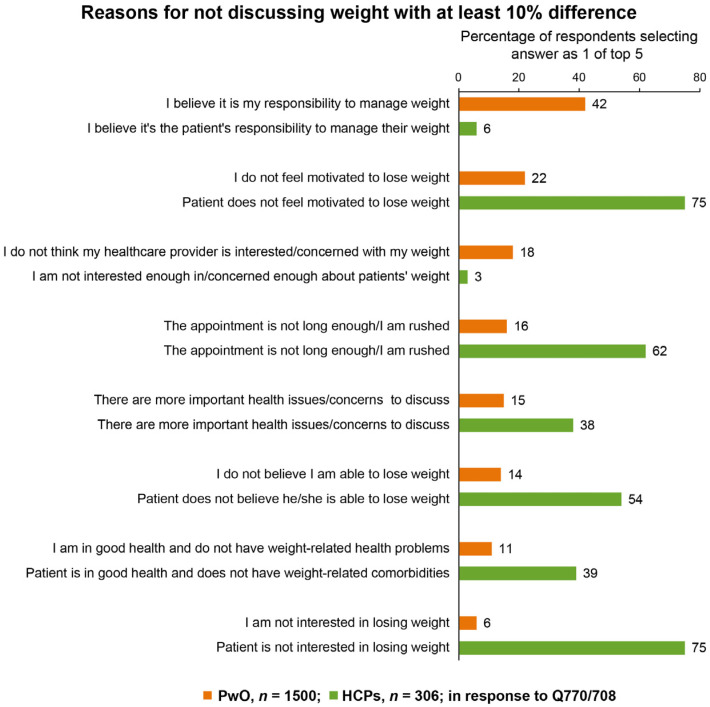
Reasons for not discussing weight with an HCP (PwO, orange) or patient (HCPs, green) with at least 10% difference. Respondents selected their top 5 reasons from the list of options. Reasons for not discussing weight with an HCP or patient with at least 10% difference between PwO and HCPs are presented. See Supplementary Figure S2 for all reasons. HCP, healthcare professional; PwO, people with obesity.

Overall, most PwO (~75%) expressed a desire for the HCP to initiate a weight management dialogue. The main reason for the HCPs initiating a dialogue was related to the presence of complications associated with obesity (selected by 71% of HCPs), followed by the patient’s BMI value as a reason for initiating dialogue compared with the global dataset (selected by 65% of HCPs). The most common reason (42%) given by PwO to not discuss their weight was the conviction that weight loss was entirely their own responsibility (Figure 4 and Supplementary Figure S2). In contrast, for HCPs, it was the perception that the patient is not interested in or not motivated for weight loss (75% each), which was in clear disagreement with the opinion of the PwO (Figure 4 and Supplementary Figure S2). Additionally, the short duration of time for each patient consultation was another of the relevant causes for the HCP not initiating a dialogue (62%).

### 3.6. Treatment Objectives

Even though 83% of PwO consider that a loss of 5–10% body weight would be beneficial to their health, 50% of PwO set themselves a weight reduction goal of 11–20% (overall average of 14.5% for all PwO). People with obesity reported that the recommendation indicated by their HCP was of a similar magnitude (mean 17%). For PwO, the weight management goals most frequently selected were to reduce the risk of obesity-related comorbidities (49%) and to improve their physical appearance (36%); the main motivators for losing weight included wanting to feel better physically (52%), to be more fit/in better shape (48%), and to improve general health (36%). The therapeutic objectives set with HCPs were mainly aimed at improving health (35%) more than physical appearance (15%).

### 3.7. Diagnosis of Obesity and Scheduling of Follow-Up Appointments

Of the PwO who had discussed excess weight or losing weight with their HCP in the last 5 years, only 66% had been formally diagnosed with obesity (44% of PwO in total), and only 36% indicated that they had any scheduled follow-up on their progress (24% of PwO in total; Figure 3a). A total of 78% of those who did not have the conversation would have liked to have had a follow-up appointment scheduled. Primary care physicians were the HCP that most PwO (78%) had ever discussed their weight with, followed by nurses (30%) and endocrinologists (29%). Endocrinologists were the professionals perceived by HCPs as being the most effective at helping PwO manage their weight.

### 3.8. Perception of Efficacy and Indication of Treatments for Obesity

**Figure 5:**
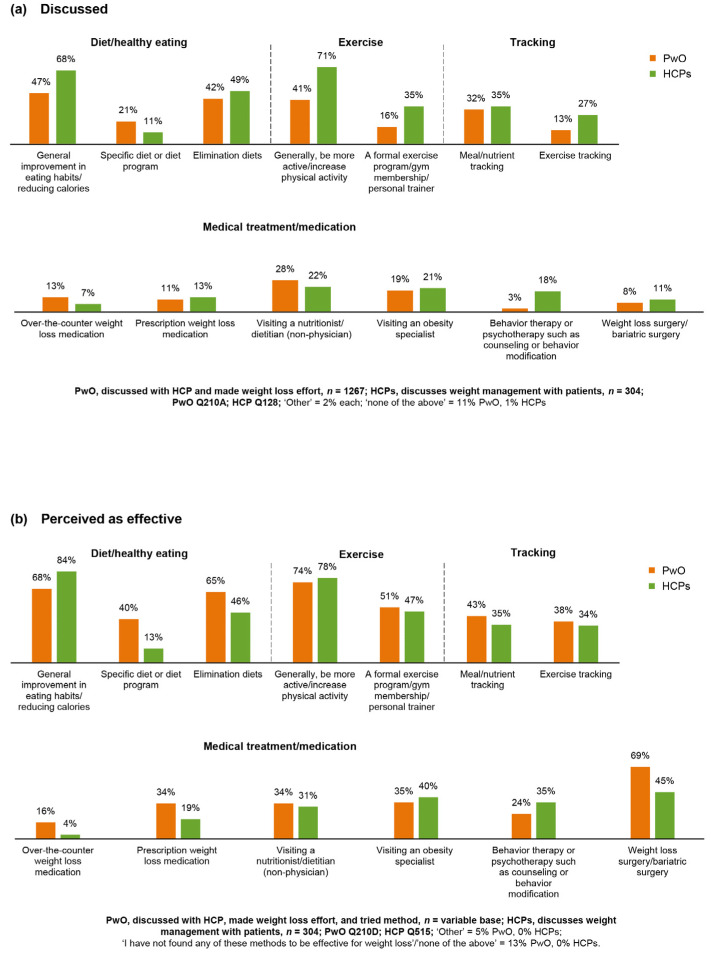
Weight management methods discussed/recommended and perceived effectiveness. (**a**) Weight management methods discussed with an HCP (reported by PwO) and recommended by HCPs; (**b**) weight management methods perceived as effective by HCPs or PwO. PwO = orange; HCPs = green. HCP, healthcare professional; PwO, people with obesity.

Both improvements in eating habits and physical activity were the strategies most commonly mentioned in conversations between PwO and HCPs (Figure 5a) and were perceived as highly effective weight management strategies by both PwO and HCPs (Figure 5b). Pharmacologic treatments were perceived to be of low efficacy by both groups. Interestingly, only 32% of HCPs believed that there are good options available for prescription weight loss medications, and 37% acknowledged that they did not know enough about prescription weight loss medications to feel comfortable prescribing them to their patients. Bariatric surgery was also considered to be a less effective mid- or long-term treatment alternative; only 39% of PwO and 55% of HCPs believed that bariatric surgery would be more effective than other treatment options for weight loss.

Of the sources of information most commonly used by PwO to manage their weight, the HCP was clearly ranked much higher than other sources of information (used by 46% of PwO), such as the internet (30%), friends and family (27%), and television programs (8%; Supplementary Figure S3).

## 4. Discussion

The Spanish results from the ACTION-IO study highlight the discrepancies between the perceptions and attitudes of PwO and HCPs; these constitute important barriers for the diagnosis and treatment of obesity per the guidelines on obesity management from the different scientific societies [14,27,28].

The study revealed a lack of recognition of obesity as a chronic disease by PwO in up to 41% of cases, even though most (83%) reported comorbidities. This lack of recognition is higher than that seen in the global dataset (32%) [24], despite fewer PwO (74%) being affected by comorbidities (ACTION-IO study steering committee, personal communication). In addition, only 26% of PwO perceived themselves as having obesity or extreme obesity, although all had a BMI of ≥30 kg/m^2^ (according to self-reported weight and height). This percentage is markedly lower than in the global population (43%; ACTION-IO study steering committee, personal communication) and from other population-based studies [29], including the National Health and Nutrition Examination Survey [30]. This misperception of body weight may help explain the reduced perception of chronic disease and delayed consultation with HCPs.

In line with the necessary identification of chronic diseases, it is alarming that only 66% of those PwO who discussed weight with their HCP received a formal diagnosis of obesity. However, this figure is higher than that observed in ACTION US (55%) [22] and ACTION Canada (48%) [23]. The low diagnosis rate could potentially explain the low rate of scheduled follow-ups in Spain (36%), which is nevertheless higher than those seen in ACTION US (24%) and ACTION Canada (28%) [22,23]. Therefore, the medical action taken differs greatly from the treatment provided for other chronic diseases, such as hypertension or diabetes mellitus, that are adequately diagnosed and monitored with the regular scheduling of follow-up appointments [31,32]. The correct diagnosis of obesity could change the perception of weight in PwO, increase engagement with HCPs, and facilitate the scheduling of follow-up appointments, enabling measures to ensure a healthy weight [33].

Among the main barriers found in the ACTION studies was the difficulties in engaging in a conversation between PwO and HCPs regarding obesity. In Spain, up to one-third of PwO do not discuss this condition with their HCPs, and this is primarily because they consider weight loss to be entirely their own responsibility, in line with previously published studies [22,23,24]. This is particularly surprising considering that the Spanish public health system provides universal coverage. Among HCPs, the most common reason for not initiating a conversation with their patients was the belief that PwO are not interested or motivated to lose weight, which differed from what was expressed by PwO themselves. Time constraints for visits were another one of the main arguments highlighted by HCPs for not initiating the dialogue. Only 19% of HCPs considered weight loss to be the sole responsibility of their patients with obesity, which is lower than the percentage reported in the global cohort (30%) [24]. Therefore, the data presented here may encourage HCPs to reflect on this matter and alter their perception that their patients lack motivation. In this regard, other studies have also shown that PwO are highly motivated, particularly when they are offered the option to participate in an intensive behavior modification program to lose weight, in which case the rate of willingness to participate reaches up to 63% [34].

The time constraints and perceptions of HCPs could explain the mean delay of 6 years observed between PwO being concerned about excess weight and the initiation of the first conversation with the HCP, which postpones the start of treatment and contributes to the development of complications. Although the mean age at the time of initial conversation with an HCP was similar in the Spanish and global ACTION-IO cohorts, it should be noted that the onset of this initial concern is more frequently under 16 years of age in Spain (20%) than in the global population of the ACTION-IO study (15%; ACTION-IO study steering committee, personal communication). This is probably a reflection of the elevated prevalence of obesity in the Spanish pediatric population, which reaches rates of 18.1% [35].

In this study, the most important objectives of PwO for losing weight were to reduce the risks associated with obesity to prevent disease, and, to a lesser extent, to improve physical appearance. Regarding the approach to therapeutic objectives, one relevant finding of the ACTION-IO study that was also observed in the Spanish data was that the weight loss goals of PwO are unrealistic, being set at losing 15% of their body weight. This figure differs from the current recommendations, which place a reduction of 5–10% as a realistic, achievable goal, which also leads to a significant decrease in the classic cardiovascular risk factors [13,36]. The desire to lose two to three times more weight than realistic goals has previously been described in other populations, including patients seeking obesity surgery [37]. This misperception of expected therapeutic performance also extends to the HCP and may contribute to feelings of frustration of PwO. All of this suggests a lack of knowledge of the reality of obesity management, which can only be improved through personal education and professional training.

In Spain, according to PwO, primary care physicians were the HCPs with whom weight was discussed most often, followed by nurses and endocrinologists. In the global dataset, after primary care physicians (63%), dietitians/nutritionists (30%) were the HCPs with whom weight was discussed most often (ACTION-IO study steering committee, personal communication). In Spain, HCPs perceived endocrinologists as the profession most effective in helping PwO, unlike in the global dataset, in which the dietitian/nutritionist was perceived as most relevant (ACTION-IO study steering committee, personal communication). These differences reflect the characteristics of the Spanish healthcare system, which offers greater access to specialists than in other countries, but with limited access to nutritionists, who mainly work in Clinical Nutrition in a hospital setting.

Improvements in dietary habits and increased physical activity were considered to be the most effective methods for weight control by PwO and HCPs. Of note was the low recommendation of prescription weight loss medication, which was mainly perceived as having low efficacy by HCPs. The ACTION-IO study, extending to the results in Spain, reveals the great lack of knowledge on the efficacy of the therapeutic options by both PwO and HCPs. Only one-third of HCPs believe that there are good pharmacologic options available for weight loss, and 37% acknowledge that they have insufficient knowledge of prescription weight loss medications. Both groups suggest that bariatric surgery is not an effective mid- or long-term treatment alternative, in stark contrast with current evidence [38]. It is therefore clear that in Spain there is a great need for education for PwO and training for HCPs in the evidence-based therapeutic management of obesity, which is a need that has been similarly identified globally [19].

Unlike in the global study, in which the internet was considered the primary source of information on all aspects related to the treatment of obesity (used by 40% of PwO; ACTION-IO study steering committee, personal communication), in Spain, PwO considered HCPs as their main source of information (used by 46% of PwO), ranking higher than other sources such as social networks and television programs. These data emphasize the importance of addressing the shortcomings of HCPs that were revealed by the study, in terms of the recognition of their patients’ motivation and objectives for weight loss and the therapeutic possibilities for this condition.

In general, the data on the Spanish population show no significant deviations from those found in the global ACTION-IO study [24]. The perception of obesity as a chronic disease, as well as the reasons for PwO not initiating a weight management dialogue with their HCP, are reasonably comparable between the ACTION US [22] and ACTION Canada [23] studies and those obtained in the Spanish population in the ACTION-IO study. The ACTION-IO Spanish data regarding the delay in starting conversations between PwO and HCPs is also comparable with the data obtained in the global ACTION-IO [24] and ACTION Canada studies [23]. Additionally, the barriers to weight loss identified by the participating Spanish PwO and HCPs were similar to the global ACTION-IO cohort [24]. Variations dependent on heterogeneity in healthcare systems can likely be improved through the development of Obesity Units and both individual and group educational resources. Coordination between endocrinologists, the leading obesity specialty in Spain, primary care physicians, dietitians, and other specialists, independent of their public or private practice, is a key element that will help to break down the barriers detected in the ACTION-IO study for the Spanish population. There is a need to invest in efforts to improve PwO awareness and greater training for HCPs whose gaps/limitations have emerged as the common denominator of the discrepancies and obstacles observed in this study. Obesity treatment requires approaches aimed at the general population, PwO, and HCPs in different areas, such as the family, school, social, or work environments, amongst others. With that in mind, it will be important for future studies to assess the perceptions, attitudes, and behaviors of a broader range of stakeholders such as nurses, dietitians, and adolescents with overweight or obesity.

The limitations of this research based on the results obtained in the Spanish population of the ACTION-IO study are identical to those of the global ACTION-IO study [24], including its cross-sectional design and the self-reported nature of the information provided by PwO and HCPs. PwO may underestimate their height and weight, which could impact on BMI estimations, and the results rely on the accuracy of the information declared retrospectively by the people surveyed. The use of the internet for the dissemination of the survey may also generate bias. Low response rates were expected and are typical of survey-based research. The proportion of Spanish HCPs who completed the survey was slightly lower, when compared with the global ACTION-IO study (6% vs. 17% internationally [24]). A higher percentage of Spanish PwO completed the survey, when compared with the global study (24% vs. 20% internationally [24]). The strengths lie in the large number of participants, the scientific quality of the questions that form part of the survey, the screening criteria applied to participants, and the stratified sampling approach to provide a representative cohort of the general population.

## 5. Conclusions

Data from the Spanish sample of the ACTION-IO study indicate that there are significant discrepancies between perceptions, attitudes, motivation, and interactions between PwO and HCPs that translate into unrealistic goals for weight loss, a low rate of obesity diagnosis, and a low number of scheduled follow-ups, all of which have been identified as obstacles to effective obesity management.

Based on these results, it is essential to increase awareness, training, and education on obesity as key elements to improve therapeutic success, quality, and the life expectancy of PwO, and thus to decrease obesity complications and healthcare expenditure. Simultaneously, the possibility of optimizing the care strategy by generating models that facilitate PwO–HCP interaction, a key element in the success of obesity treatment, should be evaluated.

## Figures and Tables

**Table 1:** Sample size considerations for the Spanish cohort of the Awareness, Care and Treatment in Obesity Management—An International Observation (ACTION-IO) study.

Country	Prevalence of Obesity *	Total Population Estimates	Estimated Number of PwO	PwO Sample Size Recommendation	Sample Size of Population with Obesity	Margin of Error ^†^
Spain	24%	47,400,000	11,376,000	1500	0.013%	2.5%

PwO, people with obesity. * Body mass index (BMI) ≥30 kg/m^2^. ^†^ PwO estimations based on the prevalence of obesity (22.9%) in the Spanish adult population, as reported by the Estudio de Nutrición y RIesgo CArdiovascular en España (ENRICA) study [7], fall within this margin of error.

**Table 2:** Sample demographics and characteristics.

	PwO (*n* = 1500)	HCPs (*n* = 306)
Age, years (range)	45 (18–81)	50 (30–69)
Gender, *n* (%)		
Female	776 (52)	218 (71)
Male	721 (48)	88 (29)
Other	3 (<1)	0
BMI classification, *n* (%)		
Respondents	1500 (100)	264 (86)
Underweight or healthy range (<25 kg/m^2^)	–	150 (57)
Overweight (25–29.9 kg/m^2^)	–	107 (41)
Obesity Class I (30–34.9 kg/m^2^)	1029 (69)	6 (2)
Obesity Class II (35–39.9 kg/m^2^)	282 (18)	0
Obesity Class III (≥40 kg/m^2^)	189 (12)	1(<1)
Number of comorbidities, *n* (%)		
0	326 (17)	–
1	370 (21)	–
2	335 (24)	–
3	242 (19)	–
≥4	227 (19)	–
HCP category, *n* (%)		
PCP	–	156 (51)
Specialist	–	150 (49)
Endocrinologist	–	73 (24)
Internal medicine (non-PCP)	–	23 (8)
Other *	–	54 (18)
Obesity specialist, *n* (%) ^†^		
Yes	–	247 (81)
No	–	59 (19)

All PwO N numbers and percentages for demographic results (age, gender) are from unweighted data, whereas the PwO percentages for non-demographic results are weighted. HCP data were not weighted, therefore, all HCP N numbers and percentages are from unweighted data. * includes HCPs who self-reported their specialty as ‘other.’ ^†^ indicates a physician who meets at least one of the following criteria: at least 50% of their patients are seen for obesity/weight management; has advanced/formal training in the treatment of obesity/weight management beyond medical school; considers themselves to be an expert in obesity/weight loss management; or works in an obesity service clinic. BMI, body mass index; HCP, healthcare professional; PwO, people with obesity; PCP, primary care physician.

## Supplementary Materials

The following are available online at https://www.mdpi.com/2077-0383/9/9/2834/s1, Figure S1: Assessing the impact of health conditions on overall health, Figure S2: Reasons for not discussing weight with an HCP, Figure S3: Sources of information used by PwO for managing weight.
