# Supplementary material for: Perceptions, Attitudes, and Barriers to Obesity Management in Spain: Results from the Spanish Cohort of the International ACTION-IO Observation Study
Source: J Clin Med. 2020 Sep 2;9(9):2834. doi: 10.3390/jcm9092834 (PMC7565674; doi:10.3390/jcm9092834)
Supplement: Supplementary file 1 [file jcm-09-02834-s001.zip › jcm-882138-supplementary.pptx]

## Slide 1
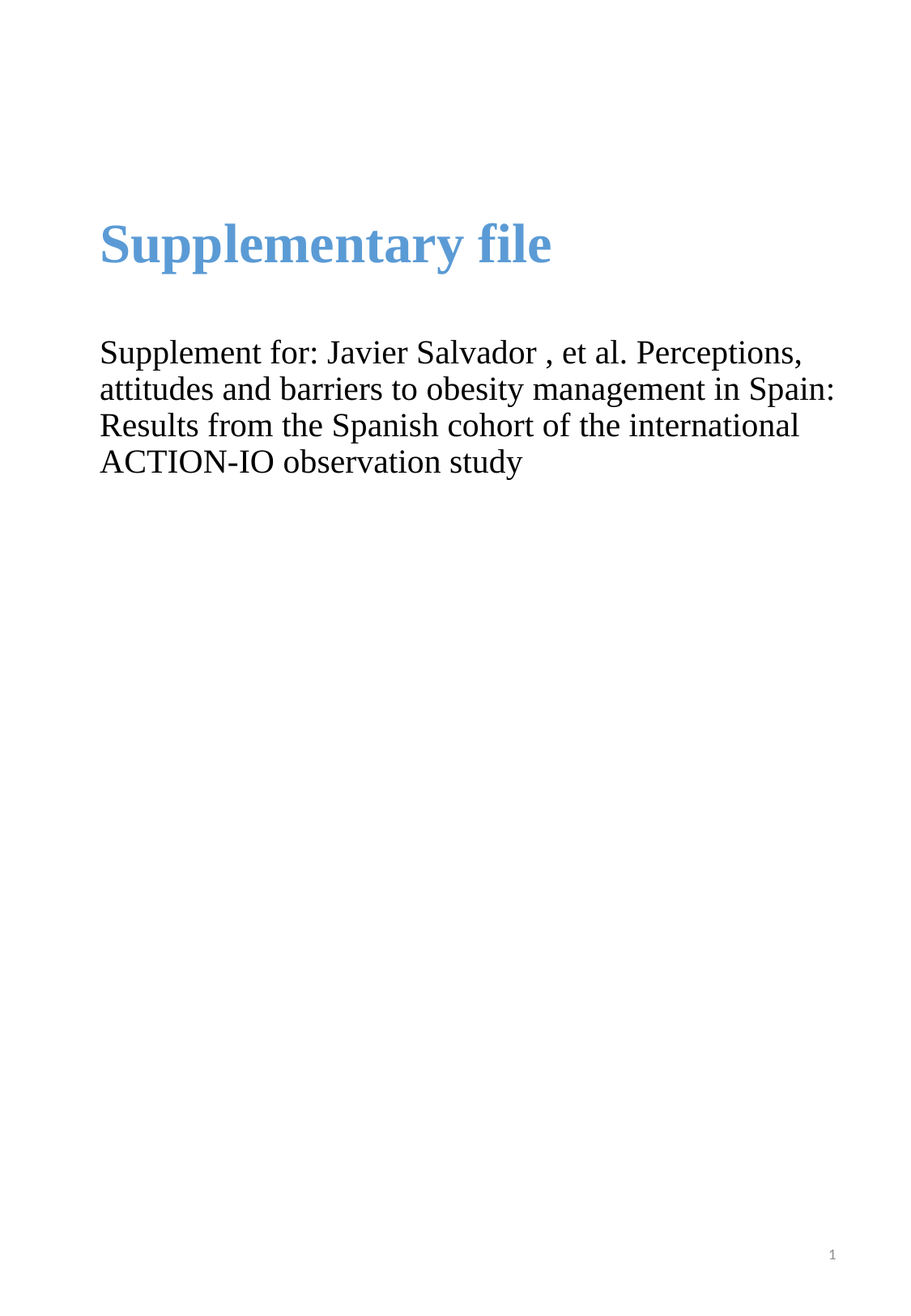

Supplementary file
Supplement for: Javier Salvador , et al. Perceptions, attitudes and barriers to obesity management in Spain: Results from the Spanish cohort of the international ACTION-IO observation study
1

## Slide 2
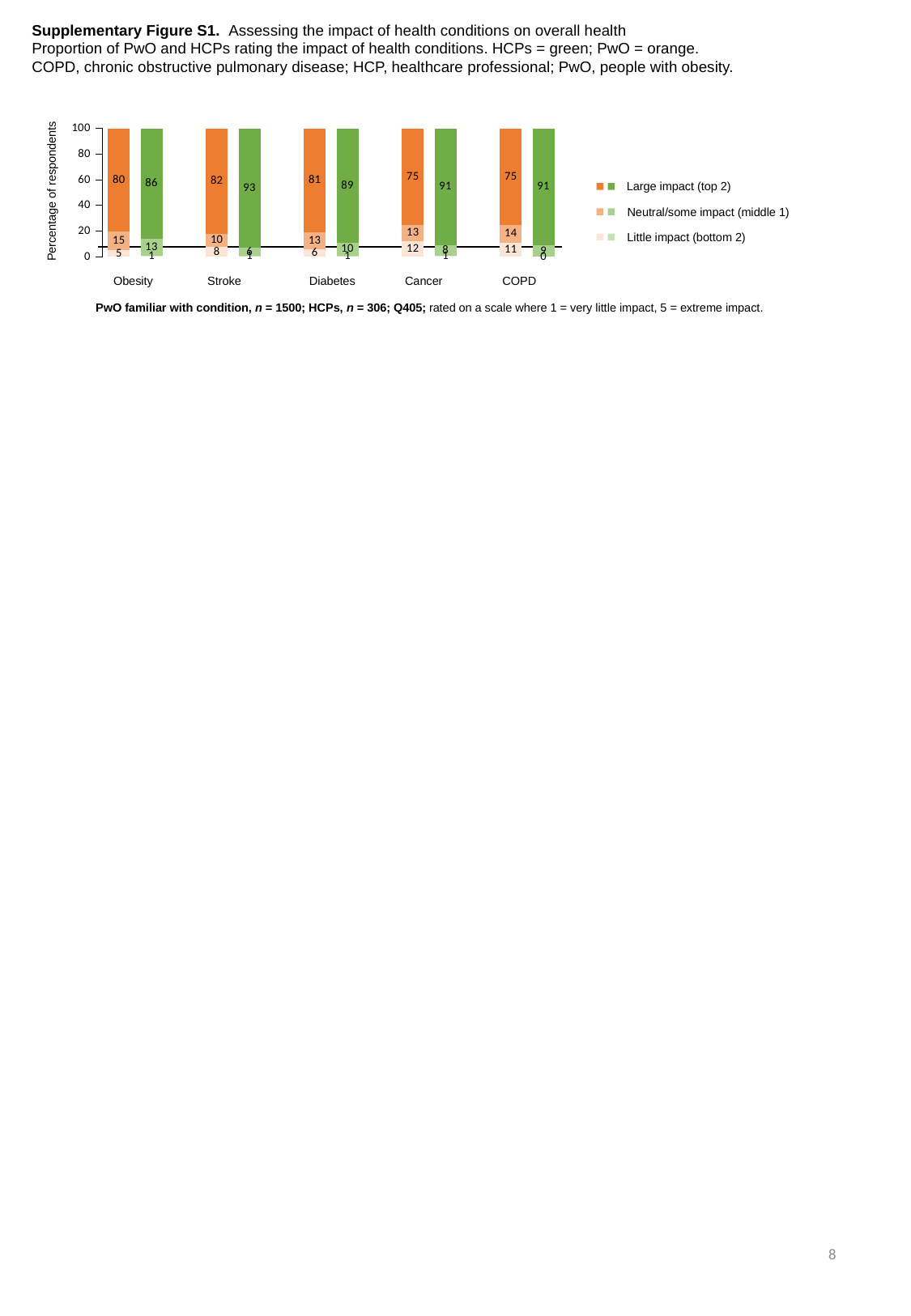

Supplementary Figure S1. Assessing the impact of health conditions on overall health
Proportion of PwO and HCPs rating the impact of health conditions. HCPs = green; PwO = orange.
COPD, chronic obstructive pulmonary disease; HCP, healthcare professional; PwO, people with obesity.
### Chart
| Category | Little impact (bottom 2) | Neutral/some impact (middle 1) | Large impact (top 2) |
|---|---|---|---|
| PwO | 5.0 | 15.0 | 80.0 |
| HCP | 1.0 | 13.0 | 86.0 |
| | None | None | None |
| PwO | 8.0 | 10.0 | 82.0 |
| HCP | 1.0 | 6.0 | 93.0 |
| | None | None | None |
| PwO | 6.0 | 13.0 | 81.0 |
| HCP | 1.0 | 10.0 | 89.0 |
| | None | None | None |
| PwO | 12.0 | 13.0 | 75.0 |
| HCP | 1.0 | 8.0 | 91.0 |
| | None | None | None |
| PwO | 11.0 | 14.0 | 75.0 |
| HCP | 0.0 | 9.0 | 91.0 |Large impact (top 2)
Neutral/some impact (middle 1)
Little impact (bottom 2)
Percentage of respondents
Obesity
Stroke
Diabetes
Cancer
COPD
PwO familiar with condition, n = 1500; HCPs, n = 306; Q405; rated on a scale where 1 = very little impact, 5 = extreme impact.
8

## Slide 3
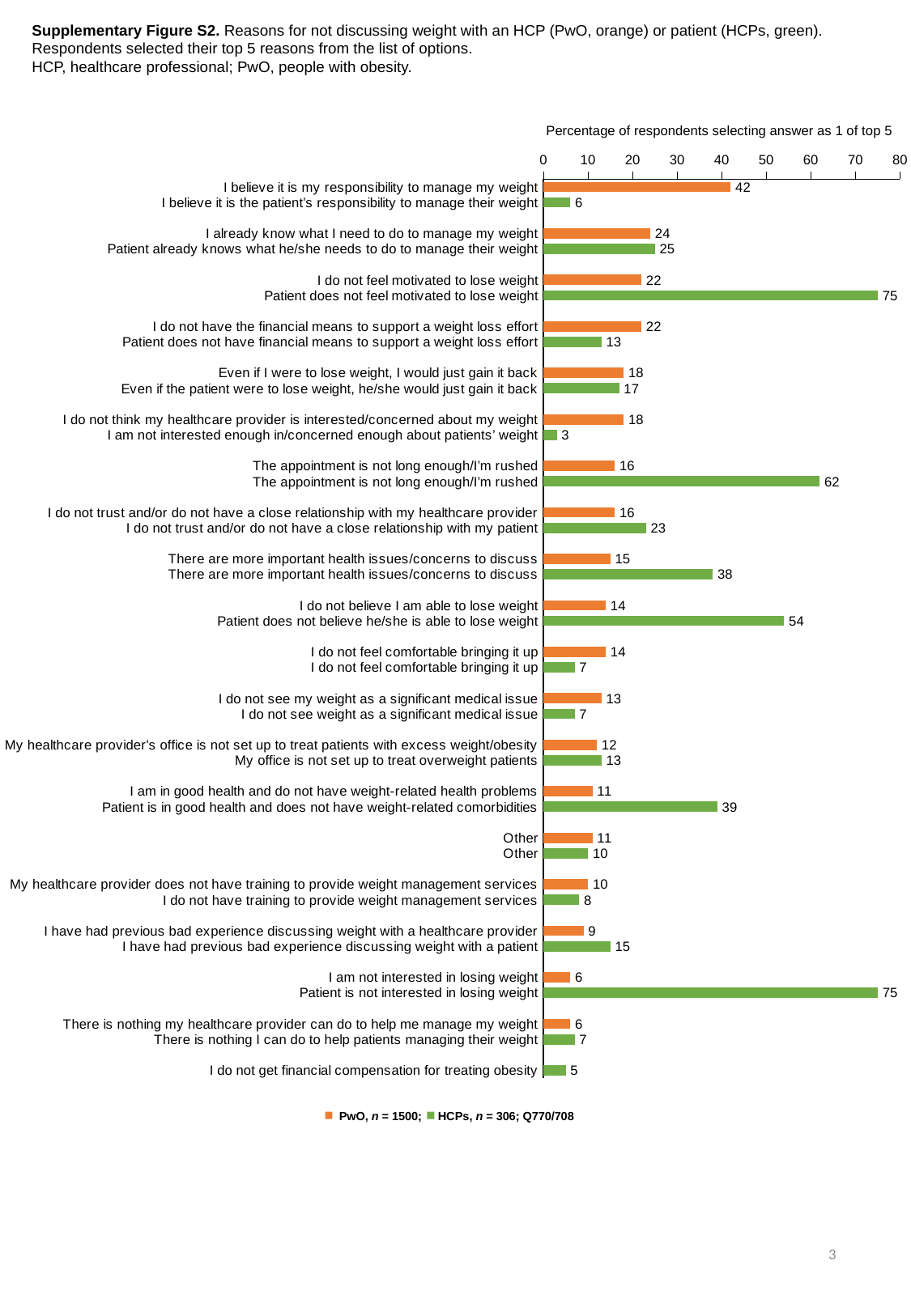

Supplementary Figure S2. Reasons for not discussing weight with an HCP (PwO, orange) or patient (HCPs, green).
Respondents selected their top 5 reasons from the list of options.
HCP, healthcare professional; PwO, people with obesity.
### Chart
| Category | |
|---|---|
| I believe it is my responsibility to manage my weight | 42.0 |
| I believe it is the patient’s responsibility to manage their weight | 6.0 |
| | None |
| I already know what I need to do to manage my weight | 24.0 |
| Patient already knows what he/she needs to do to manage their weight | 25.0 |
| | None |
| I do not feel motivated to lose weight | 22.0 |
| Patient does not feel motivated to lose weight | 75.0 |
| | None |
| I do not have the financial means to support a weight loss effort | 22.0 |
| Patient does not have financial means to support a weight loss effort | 13.0 |
| | None |
| Even if I were to lose weight, I would just gain it back | 18.0 |
| Even if the patient were to lose weight, he/she would just gain it back | 17.0 |
| | None |
| I do not think my healthcare provider is interested/concerned about my weight | 18.0 |
| I am not interested enough in/concerned enough about patients’ weight | 3.0 |
| | None |
| The appointment is not long enough/I’m rushed | 16.0 |
| The appointment is not long enough/I’m rushed | 62.0 |
| | None |
| I do not trust and/or do not have a close relationship with my healthcare provider | 16.0 |
| I do not trust and/or do not have a close relationship with my patient | 23.0 |
| | None |
| There are more important health issues/concerns to discuss | 15.0 |
| There are more important health issues/concerns to discuss | 38.0 |
| | None |
| I do not believe I am able to lose weight | 14.0 |
| Patient does not believe he/she is able to lose weight | 54.0 |
| | None |
| I do not feel comfortable bringing it up | 14.0 |
| I do not feel comfortable bringing it up | 7.0 |
| | None |
| I do not see my weight as a significant medical issue | 13.0 |
| I do not see weight as a significant medical issue | 7.0 |
| | None |
| My healthcare provider's office is not set up to treat patients with excess weight/obesity | 12.0 |
| My office is not set up to treat overweight patients | 13.0 |
| | None |
| I am in good health and do not have weight-related health problems | 11.0 |
| Patient is in good health and does not have weight-related comorbidities | 39.0 |
| | None |
| Other | 11.0 |
| Other | 10.0 |
| | None |
| My healthcare provider does not have training to provide weight management services | 10.0 |
| I do not have training to provide weight management services | 8.0 |
| | None |
| I have had previous bad experience discussing weight with a healthcare provider | 9.0 |
| I have had previous bad experience discussing weight with a patient | 15.0 |
| | None |
| I am not interested in losing weight | 6.0 |
| Patient is not interested in losing weight | 75.0 |
| | None |
| There is nothing my healthcare provider can do to help me manage my weight | 6.0 |
| There is nothing I can do to help patients managing their weight | 7.0 |
| | None |
| I do not get financial compensation for treating obesity | 5.0 |Percentage of respondents selecting answer as 1 of top 5
PwO, n = 1500; HCPs, n = 306; Q770/708
3

## Slide 4
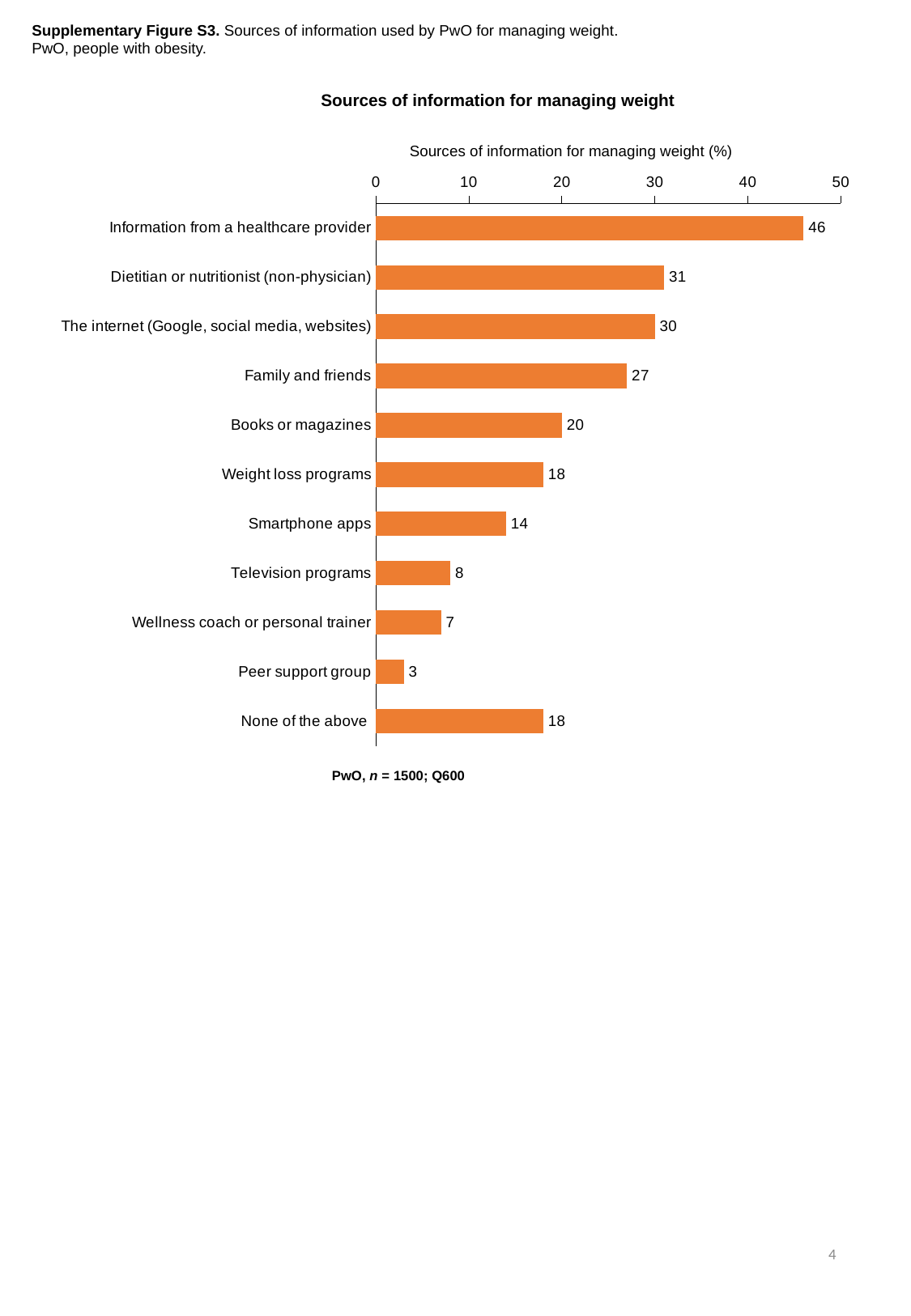

Supplementary Figure S3. Sources of information used by PwO for managing weight.
PwO, people with obesity.
Sources of information for managing weight
Sources of information for managing weight (%)
### Chart
| Category | |
|---|---|
| Information from a healthcare provider | 46.0 |
| Dietitian or nutritionist (non-physician) | 31.0 |
| The internet (Google, social media, websites) | 30.0 |
| Family and friends | 27.0 |
| Books or magazines | 20.0 |
| Weight loss programs | 18.0 |
| Smartphone apps | 14.0 |
| Television programs | 8.0 |
| Wellness coach or personal trainer | 7.0 |
| Peer support group | 3.0 |
| None of the above | 18.0 |PwO, n = 1500; Q600
4
